# Supplementary material for: Super-diffusion of excited carriers in semiconductors
Source: Nat Commun. 2017 May 11;8:15177. doi: 10.1038/ncomms15177 (PMC5437287; doi:10.1038/ncomms15177)
Supplement: Supplementary Information — Supplementary Figures [file ncomms15177-s1.pdf]

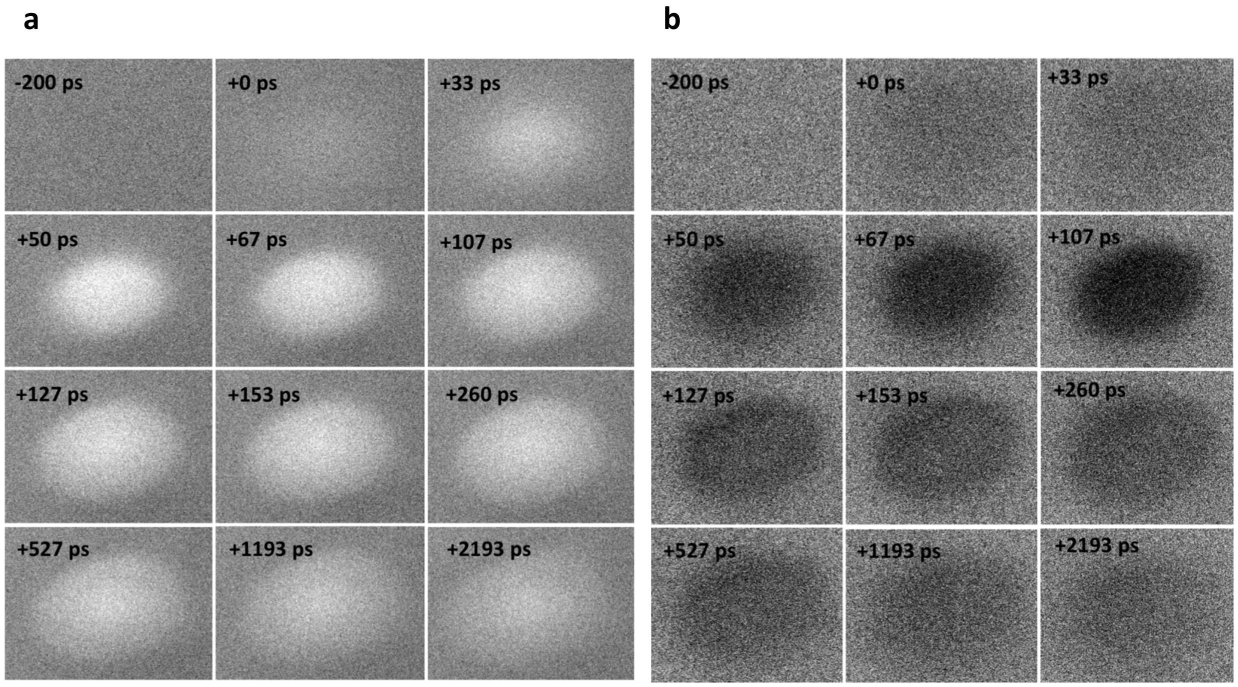

Supplementary Figure 1. Scanning ultrafast electron microscopy (SUEM) images of the dynamics of (a) electrons and (b) holes in p-type and n-type silicon, respectively. These SUEM images complement those in Fig. 2 of the main text. Here, the dynamics is shown at the same time delays for electrons and holes.

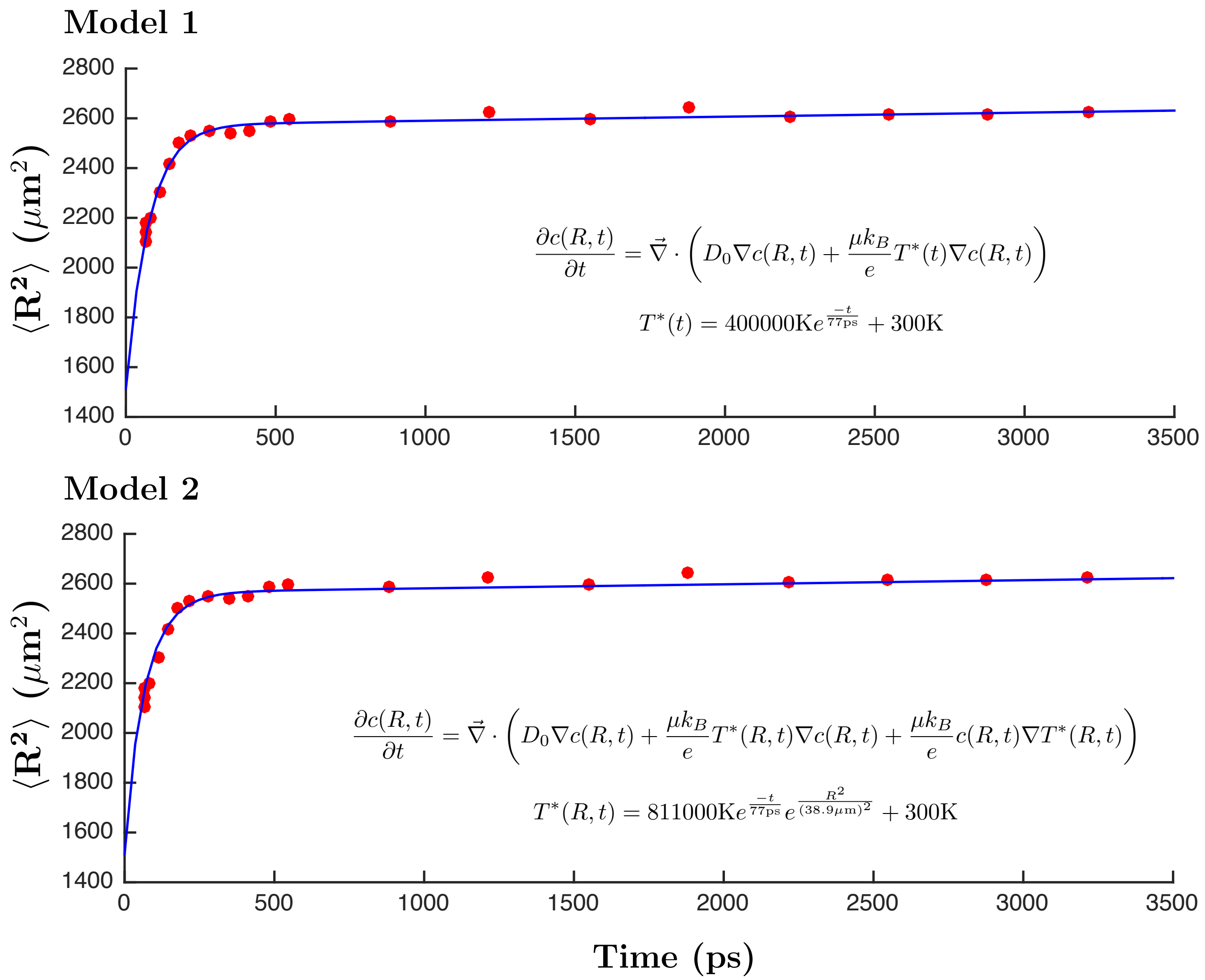

Supplementary Figure 2. Time evolution of the second moment (see eq. 1 in the main text) for a uniform temperature distribution (top panel) and a non-uniform temperature distribution with a gaussian spatial profile (bottom panel). Using the same relaxation time of 77 ps and initial effective temperatures within a factor of 2 of each other, both models give excellent agreement with experiment. Hence the choice of using a constant temperature, as done in the main text, does not affect the interpretation of the results. The differential equations solved for the two models, which combine eq. 3 and eq. 4 in the main text, are also given in the figure.
